# Sunshine on the South Coast

**Published:** 1896-02-29

**Authors:** 


### Sunshine on the South Coast.

There is but little doubt that English south coast watering-places suffer in consequence of the general tendency to estimate the value of a health resort according to its fitness as a sanatorium for consumption, a very mistaken criterion, for it cannot honestly be said that any one of them is an ideal spot for the treatment of cases suffering from that disease. To those, no doubt, who come from cold clay districts or from large cities with their fogs and want of light, or from so-called bracing but too often killing districts in the North of England, a winter at some of our south-western health resorts, if not the best that can be done, is at least an immense improvement on staying at home. Still, it is not the best possible. In Egypt, Algiers, some parts of the Riviera, and especially in certain high altitudes in the Alps, places are to be found in which the climatic treatment of phthisis can be carried on with much greater chance of success, but it is obviously unfair to gauge the general utility of a health resort by its efficacy in the treatment of one particular class of disease.

No doubt, in times when travelling was more difficult, accommodation less comfortable, and wealth less widely spread, the fact that few left home in winter except from urgent necessity, and that such necessity mostly originated in some disorder of the chest, led to this criterion being set up. Now, however, things are different. A far larger number of people have it in their power to winter away from home, and a far larger area is open to them to select from in their choice of a place of shelter; and thus, while rich people in the hopeful stages of phthisis are tempted further afield, a large number of real invalids, both of the wealthy and the middle classes of society, are able to winter at such places as Bournemouth or Ventnor or Torquay amid such an amount of sunshine and fresh air as, if not so curative as in more happy climates, at least helps greatly to hold the enemy at bay.

Walking, however, on the cliffs at Bournemouth in the keen, cold air, and bathed in the bright sunshine which has prevailed there, as at other places on the same coast, during the last few days, it has been impossible to hide from oneself that the true therapeutic uses of this sunny coast extend far beyond diseases of the chest, and apply especially to cases in which no gross disease at all is present, at any rate, so far as disease is discoverable by stethoscope or eye. Cases of anæmia, of debility, of apparently causeless "breakdown," neurasthenia, if one likes to call it so, require not only complete change of surroundings, but also a large modicum of fresh air and sunshine, all of which they may get, even in winter, at the places we refer to. It is all very well to call debility "nervous," and to ridicule indecision and timidity as showing mere want of "will." But such things are real, and once they have got hold are far from easy to shake off. Doubtless, drugs are useful. The iron tonics with which so many white-faced girls are drenched, the pill and draught by which the harassed city man drives on his torpid liver and revives his wits, even the valerian and other anti-spasmodics by which so many nervous women are roused for a time to a due perception of the fitness of things, all are good in their way, but their way is not for long. The patient, left amid the old surroundings which have caused the old condition of things, falls back into it again, the second state too often being worse even than the first. All these people require to be lifted out of the circumstances amid which they have fallen ill. In summer there is no difficulty; in winter we find in the treatment of such cases the real utility of the south coast as a sanatorium. And so it is being used. Everywhere one sees large hotels and comfortable pensions in which, at some expense undoubtedly, but at little trouble, people who are out of sorts can lead a healthy and well-protected life, mixing with fresh people, doing fresh things, riding, bicycling, and golfing, getting as much fresh air as possible, and eating as much as, or perhaps a little more than, they can digest. The easy access, large hotel accommodation, and the climatic advantages of the watering-places on the south coast of England give them a large and ever-growing importance in the winter treatment of many conditions which are purely functional, besides being havens of shelter for diseases which are organic.

